# Optimal preparation of SARS-CoV-2 viral transport medium for culture

**DOI:** 10.1186/s12985-021-01525-z

**Published:** 2021-03-10

**Authors:** Julie McAuley, Claire Fraser, Elena Paraskeva, Elizabeth Trajcevska, Michelle Sait, Nancy Wang, Eric Bert, Damian Purcell, Richard Strugnell

**Affiliations:** 1 Department of Microbiology and Immunology, The University of Melbourne at the Peter Doherty Institute for Infection and Immunity, Melbourne, 3000 Australia; 2 Media Production Unit, Department of Microbiology and Immunology, The University of Melbourne at the Peter Doherty Institute of Infection and Immunity, Melbourne, 3000 Australia; 3 Microbiological Diagnostic Unit, Department of Microbiology and Immunology, The University of Melbourne at the Peter Doherty Institute of Infection and Immunity, Melbourne, 3000 Australia; 4 3DMeditech, 44 Cook Street, Port Melbourne, 3207 Australia

**Keywords:** SARS-CoV-2, Culture, Transport media, VTM, Diagnosis

## Abstract

**Introduction:**

The sudden arrival of the COVID-19 pandemic placed significant stresses on supply chains including viral transport medium (VTM). The VTM that was urgently required needed to support viral replication, as well as other routine diagnostic approaches. We describe the preparation and validation testing of VTM for rapidly expanding diagnostic testing, where the capacity of the VTM to preserve viral integrity, for culture, isolation and full sequence analysis, was maintained.

**Methods:**

VTM was prepared using different methods of sterilization then ‘spiked’ with virus. The VTM was investigated using viral culture in Vero cells, and for nucleic acid detection by quantitative PCR.

**Results:**

The best results were obtained by filter and autoclave-based sterilization. The VTM proved robust for culture-based analyses provided the inoculated VTM was stored at 4 °C, and tested within 48 h. The filtered VTM also supported PCR-based diagnosis for at least 5 days when the mock inoculated VTM was held at room temperature.

**Discussion:**

The manual handling of VTM production, including filling and sterilization, was optimized. SARS-CoV-2 was spiked into VTM to assess different sterilization methods and measure the effects of storage time and temperature upon VTM performance. While most diagnostic protocols will not require replication competent virus, the use of high quality VTM will allow for the next phase of laboratory analysis in the COVID-19 pandemic, including drug and antibody susceptibility analysis of re-isolated SARS-CoV-2, and for the testing of vaccine escape mutants.

## Key Points

- The disruption by COVID-19 to international supply chains has made importation of diagnostic reagents from traditional sources more complex, and self-reliance is becoming important
- COVID-19 diagnosis generally relies on detecting the virus through nucleic acid amplification, or antigen identification
- Though viral culture has become a `lost art', culture will be necessary to support nucleic acid-based diagnosis with culture to resolve breakthrough infections in those vaccinated, treated or seemingly immune
- This paper examines the ability of viral transport medium to support COVID-19 viral culture

## Introduction

The COVID-19 global pandemic has tested the supply chains that support the diagnosis of infections, including testing equipment and assay components and the availability of swabs and viral transport medium (VTM), in some areas, became limiting for COVID-19 testing.

The CDC has a draft Standard Operating Procedure (SOP) for the preparation of VTM (SOP#:DSR-052-04) and VTM must sustain viral integrity and suppress contaminating microorganisms that might interfere with diagnosis [1]. In Australia, the Therapeutic Goods Administration requests VTM sterility and a composition consistent with the CDC SOP.

The Media Production Unit (MPU) at the Peter Doherty Institute for Infection and Immunity, a small accredited (ISO/IEC17025:2017), near at-cost, media provider for local and regional diagnostic laboratories using largely manual processes, was asked to supply VTM at 20,000 units per week. Production of VTM at the MPU expanded in parallel with the local production of 3D-printed nasopharyngeal swabs to address increased testing [2]. This study analyses sterilization and VTM ability to support to the growth of SARS-CoV-2 in permissive cells.

## Materials and methods

### Virus

Stocks of SARS-CoV-2 isolate hCoV-19/Australia/VIC01/2020 [3] was produced as previously described [4]. The undiluted stock had a 50% Tissue Culture Infectious Dose (TCID_50_) of 10^5.24^ TCID_50_/mL.

### Media

VTM was produced according to CDC specifications, as a 3 mL volume in a 10 mL plastic, sterile tube., comprising Anderson’s modified Hanks Balanced Salt Solution (8.0 g/L NaCl, 0.4 g/L KCl, 0.05 g/L Na_2_HPO_4_, 0.06 g/L KH_2_PO_4_, 1.0 g/L Glucose, 0.7 g/L NaHCO_3_, 0.2 g/L MgSO_4_.7H2O, 0.14 g/L CaCl_2_.2H_2_O) with 2% v/v heat-inactivated fetal bovine serum (Sigma, Australian origin), 100 µg/mL gentamicin and 0.5 µg/mL amphotericin B (https://www.cdc.gov/coronavirus/2019-ncov/downloads/Viral-Transport-Medium.pdf). The VTM was sterilized by autoclaving (110 °C for 10 min), unfiltered but gamma-irradiated (15 kGy), or filter-sterilized (0.2 µm). In viral infection studies, minimal essential medium (MEM) (Sigma, Mannheim, Germany), supplemented with 10μMHEPES, 2 mM glutamine and antibiotics ((100 units/mL Penicillin G, 100 µg/mL Streptomycin), but without the presence of fetal bovine serum, was used.

### Antimicrobial properties of VTM prepared by different means

Bacterial or yeast stationary phase cultures were diluted and inoculated into VTM at 10^3^–10^4^ CFU. The VTM was incubated for 3 days at RT (18–20 °C), whereafter 10µL was removed and plated onto HBA. Growth on HBA was compared with the inoculum and reported as no growth (NG), < 10 colonies ( +), or > 300 colonies (+ + +).

### Virus ‘spiking’ experiments

The ‘spiking’ of VTM with SARS-CoV-2 was conducted in the Doherty Institute PC3 Laboratory. Each 3 mL VTM sample (n = 3 per preparation condition) was incubated with 300µL of SARS-CoV-2 stock (at 10^5.25^TCID_50_/mL) to yield 60,000 infectious viral particles per 3.3 mL. Samples were incubated at RT (18–19 °C, 40% relative humidity) or at 4 °C, for various periods before 200µL was harvested and a TCID_50_ performed on each sample. For incubation conditions at 25 °C, 37 °C and 45 °C, samples in tubes were placed in sealed containers, then incubated in separate tissue culture incubators (5%CO_2_) specifically set to each temperature.

### 50% Tissue Culture Infectious Dose assay (TCID_50_)

Tissue culture plates (24 well) were seeded with Vero cells 24 h prior to use and were used at ~ 95% confluency. The samples were serially diluted and inoculated (4 replicates/sample and up to 4 serial dilutions) and incubated for 45 min to enable virus infection of monolayers, then infection media (without FBS) but with TPCK trypsin (1 µg/mL) was added. The plates were incubated (37 °C, 5% CO_2_) and examined 3 d post-inoculation for virus-induced cytopathic effect (CPE). The TCID_50_/mL was calculated [5] and plotted as the mean ± standard error of repeated experiments. A two-way ANOVA with Tukey post-tests and *p* < 0.05 was considered to indicate statistical significance.

### Quantitative PCR assay of virus in VTM

Inactivated SARS-CoV-2 virus was inoculated into 3 mL of filtered sterilized VTM or Kang-Jian Viral transport media (catalogue no KJ502-19) and tested at day 0 then 5 days post inoculation after storage at RT or 4 °C. A 300 µl sample was extracted on the Chemagic360 platform using a Chemagic Viral Nucleic Acid Kit (Perkin Elmer) and eluted into 60µL. PCR targeting the SARS-CoV-2 E gene comprised 5µL nucleic acid extract, 1 × qScript XLT One-Step RT-qPCR ToughMix Low ROX (Gene Target Solutions), 400 nM of primers E_Sarbeco_F and E_Sarbeco_R and 200 nM probe E_Sarbeco_P1 [6] using conditions described by Corman et al. 2020 [6].

## Results

The antimicrobial properties of the VTMs produced by the three sterilization methods is shown in Table 1. The antifungal activity of the VTM was decreased by autoclaving or gamma irradiation, whereas the antibacterial activity was not affected by the method of preparation.

**Table 1:** Inhibition of bacteria and yeasts by VTM

Organism	Inoculum	Filtered	Autoclaved	γ-irradiated
*Streptococcus pyogenes*	+++	NG	NG	NG
*Escherichia coli*	+++	NG	NG	NG
*Staphylococcus aureus*	+++	NG	NG	NG
*Candida albicans*	+++	NG	+++	+++
*Saccharomyces cerevisiae*	+++	NG	+	+++

Stationary phase cultures of 3 bacteria and 2 yeasts were diluted and inoculated into VTM with concentrations between 10^3^ and 10^4^ bacteria or yeast, mixed and 10 µl was immediately removed and plated onto horse blood agar (Inoculum). The VTM was then incubated for 3 days at RT (18–20 °C) whereafter 10ul was removed and plated onto HBA. The growth on HBA was determined. No growth (NG), +  < 10 colonies, +++  > 300

**Fig. 1:**
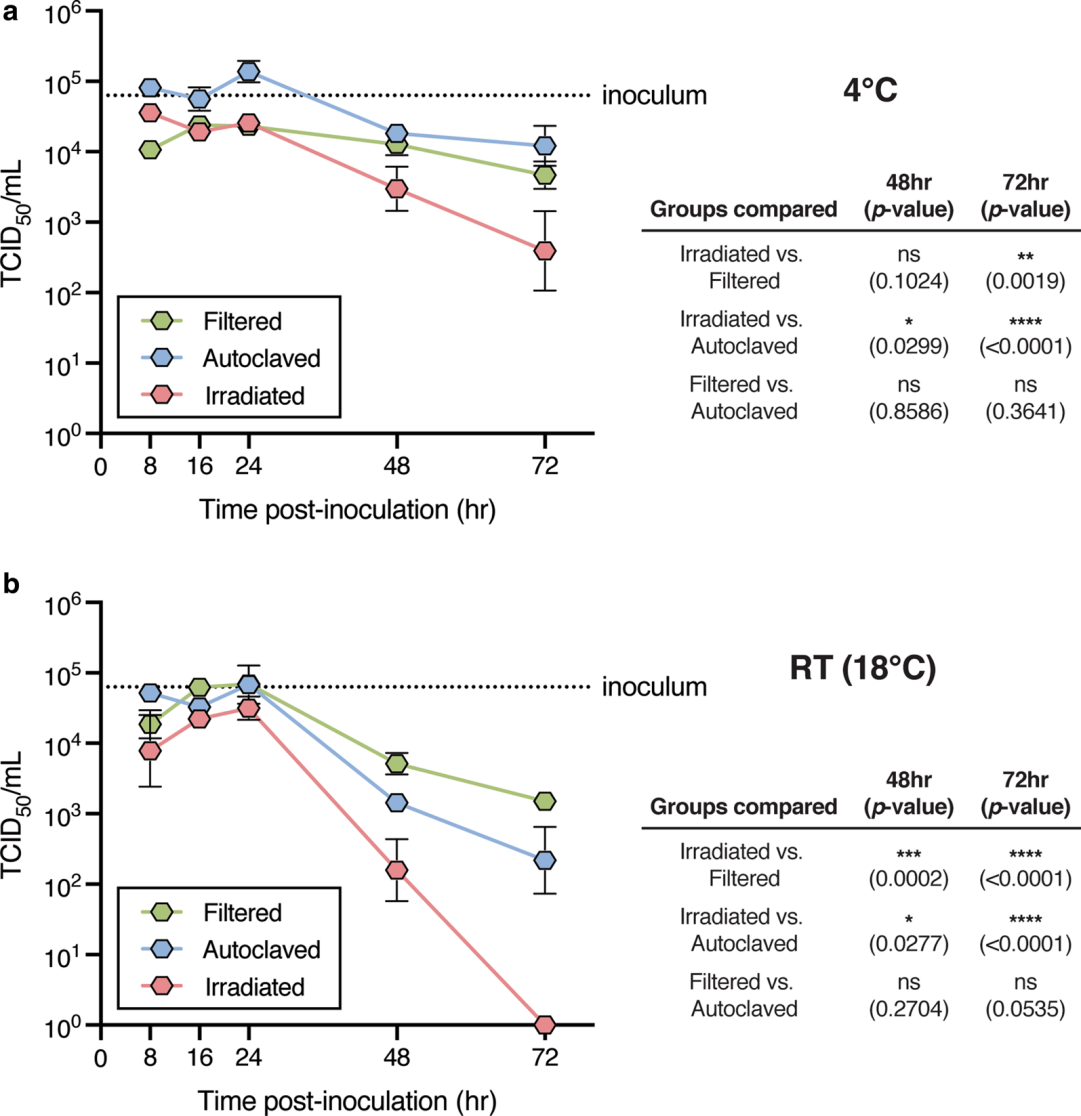
Recovery of replicative virus from VTM. VTM was prepared using three methods—filter sterilization, sterilization by autoclaving, or by gamma-irradiation. The VTM was inoculated with replicative virus then stored for various periods of time at 4 °C (**a**) or RT (18–20 °C, (**b**)) before viable virus was assayed using Vero cells, serially diluted VTM, where viral cytopathic effect (CPE) was examined. The virus recovered was expressed as TCID_50_/mL. At each time point, the data for each method of preparation was compared using a Two ANOVA and Tukey post-test correction. A *p* < 0.05 was considered significant (ns: not significant)

Approximately 10^4.8^ TCID_50_ SARS-CoV-2 virus was seeded into the VTM. The samples were stored at RT (18–19 °C), or at 4 °C for 8–72 h. After storage, dilutions of VTM were inoculated onto 95% confluent Vero cells and CPE determined after 3 days. The TCID_50_/mL was calculated for each sample and compared with the starting inoculum (Fig. 1). Samples stored at RT or 4 °C showed relatively similar infectious viral loads over 24 h, where after the viral load in the VTM prepared by gamma irradiation declined significantly.

Samples stored at 18 °C revealed limited impact on recovery of replicative virus with storage over the first 24 h. After 24 h, there was a significant decline in recovered virus from VTM that was prepared by gamma-irradiation, and by 48 h at RT, no replicative virus was recovered from VTM that was prepared by gamma-irradiation.

**Fig. 2:**
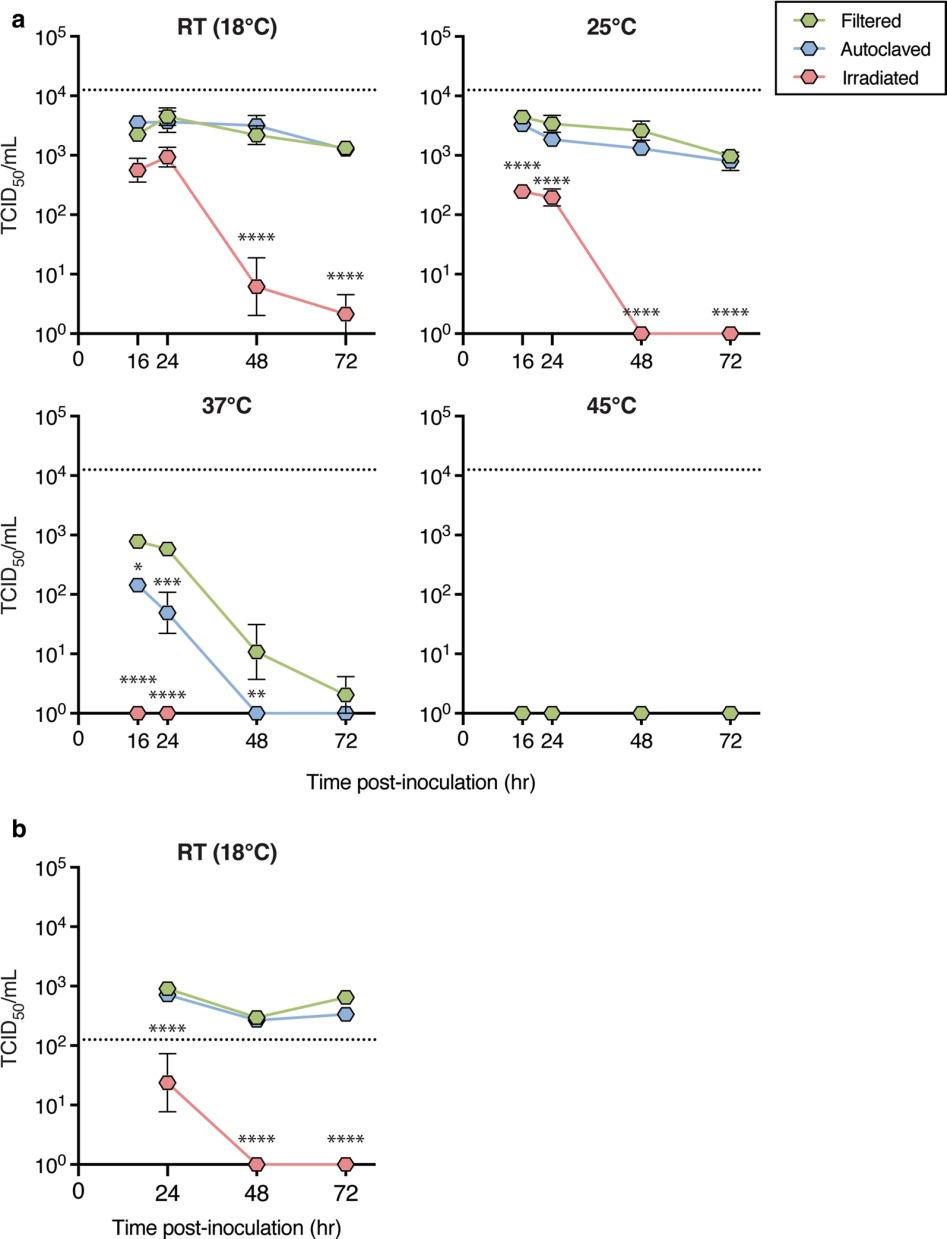
Preservation of viral infectivity in samples varies with VTM sterilization method and is affected by storage temperature and starting dose. VTM sterilized by filtration, autoclave or irradiation was inoculated at a level of **a** 1.2 × 10^4^ TCID_50_/mL or **b** 1.2 × 10^2^TCID_50_/mL SARS-Cov-2, and then incubated at indicated temperatures for up to 72 h. Shown are mean ± SEM for 5–7 data points, pooled from two independent experiments. Dashed lines represent the final concentration of virus used to inoculate the VTM. Two-way ANOVA with Tukey’s post-tests was used to compare all groups at each time point; * indicates any group that showed a *p* value less than 0.05 compared to the filtered group, * *p* < 0.05, **p < 0.01, ***p < 0.001, ****p < 0.0001

Given that many COVID-19 samples may be stored and/or transported under suboptimal conditions, the effect of storage temperature on viral recovery was further examined (Fig. 2a). Irradiated VTM inoculated with 25,000 TCID_50_ SARS-Cov-2 retained some infectivity for Vero cells over the first 24 h when stored at 18 °C or 25 °C, but not after this period. Inoculated samples stored at 37 °C yielded lower levels of infectious virus. VTM sterilized by autoclaving or filtration retained full infectivity for 48 h at 18 °C or 25 °C, but infectivity declined after 24 h at 37 °C. The inoculum was inactivated by sample storage at 45 °C, regardless of VTM preparation method, and length of incubation.

The effect of low virus load on replicative viral recovery was examined. To test the effect of VTM preparation on low dose inocula, SARS-CoV-2 was seeded at 250 TCID_50_ /mL in filtered, autoclaved or irradiated VTM and stored at room temperature, with samples taken every 24 h (Fig. 2b). The starting inocula yielded 250 infectious units/mL, the level of which were sustained by filtered or autoclaved VTM for at least 72 h stored at 18 °C. Under these conditions, infectious virus was recovered from two of six samples inoculated into irradiated VTM at 24 h (where the infectious viral load was significantly reduced in the positive samples), but no virus was recovered after 24 h from inoculated irradiated VTM stored at 18 °C. To confirm that the filtered VTM did not interfere with PCR-based diagnosis of COVID-19, inactivated SARS-CoV-2 was added to VTM (MPU or Kan-Jian (control)) and incubated for 5 days at RT, or at or 4 °C, prior to qPCR testing (Table 2). The Ct values for all 6 samples, day 0 and day 5 at RT or 4 °C, were within the expected ranges (27.1–28.2).

**Table 2:** Ability of VTM to support qPCR

		Swab held at 4 °C		Swab held at 18–20 °C	
Media	Period	0 days	5 days	0 days	5 days
Kang-jian	Ct Value	ND	28.0	27.1	28.0
MPU VTM	Ct Value	ND	27.8	28.2	28.0

Quantitative PCR (qPCR) was used to analyse inactivated SARS-CoV-2 was ‘spiked’ into commercially source viral transport media (Kang-Jian) or VTM produced by the Media Production Unit. The transport media were held at 4 °C or RT (18–20 °C) for 5 days, because nucleic acid was extracted and the numbers of genomes determined by cycle threshold (Ct) number in the PCR. The values were within normal variation (i.e. one cycle, 27.5–28.5 cycles) for the amount of nucleic acid inoculated into the transport media. ND: not done

## Discussion

COVID-19 drove the parallel development of diagnostic regimes on different platforms, using assays that detect viral nucleic acid, viral proteins and antibodies induced by infection. As therapies and vaccines are introduced, it will be important to isolate infection-competent virus from diagnostic samples to test for e.g. virus ‘escape mutants’ to complement qPCR testing. This study explores the ability of VTM to support SARS-CoV-2 culture, produced using different sterilization options—filter sterilization, autoclave and gamma-irradiation.

The Australian Department of Health asked that VTM comply with CDC specifications [7], despite recent evidence that, for short transits, saline performs as well at VTM [8, 9] for standard qPCR-based diagnostic procedures. We expected that culture-based analysis would require greater preservation of the virus present in diagnostic samples. Although not previously a VTM provider, the MPU was asked to provide 20,000 vials of VTM per week and sought to optimize the production so as to minimize its impact on staff and ongoing media supply operations.

Gamma-irradiated VTM was less supportive of viral culture, resulting in a drop of viral titre of between 100 and 10,000-fold, 2–3 days after inoculation, which was more marked if the samples were stored at RT. A similar result was observed when much lower levels of virus were used, or when the storage temperatures were increased. After 72 h at RT, no infectious virus was recovered from VTM prepared by gamma-irradiation, though infectious virus was found in VTM prepared by filtration or autoclaving. All VTMs failed to protect replicative virus when the samples were stored at 45 °C. The reason why irradiation would destroy the preservative properties of VTM were not explored but irradiation can denature bovine serum proteins [10]. VTM that was filter-sterilized, or autoclaved, preserved SARS-CoV-2 replication, however the antifungal present in the VTM was inactivated by autoclaving, suggesting that VTM prepared by autoclaving might be less useful where diagnostic samples are not refrigerated, or where transit time from sampling to diagnostic lab is longer than 1–2 days.

The next phase of the global response to the COVID-19 pandemic will be to more routinely isolate and sequence SARS-CoV-2 found in clinical samples, and to test whether they remain sensitive to the COVID-19 antivirals or plasma-derived [11] or laboratory-generated [12, 13] antibodies. After vaccination is implemented, SARS-CoV-2 that escape vaccine-induced immunity (14) will need to be studied and this will require viral culture. The data presented here suggests viral recovery will be optimized by the use of filter sterilized VTM and storage of inoculated VTM at or below 25 °C, for less than 72 h.

## Conclusions

The conservative preservation of SARS-CoV-2 in patient samples to enable viral culture will be required to fully understand why the growing list of COVID-19 vaccines or therapies fail. We showed that the method of CDC VTM preparation is critical to preserving the capacity of the virus to replicate. The optimal method to preserve viral culture used filter sterilization, while other methods supported nucleic acid-based diagnosis.

## Data Availability

The data contained within the manuscript is available for electronic transmission via the corresponding author.

## Abbreviations

VTM: Viral transport medium
qPCR: Quantitative detection of virus using the polymerase chain reaction
CDC: Centers for Diseases Control (US, based in Atlanta, GA, USA)
MPU: Microbiological Media Production Unit of The University of Melbourne
SARS-CoV-2: Viral coronavirus agent causing Severe Acute Respiratory Syndrome, COVID-19
TCID50: Tissue culture infectious dose 50%
HBA: Horse blood agar
CPE: Cytopathic effect due to viral infection of tissue culture cells
